# Exploring Potential Chemical Transformation by Chemical Profiling Approach for Rapidly Evaluating Chemical Consistency between Sun-Dried and Sulfur-Fumigated Radix Paeoniae Alba Using Ultraperformance Liquid Chromatography Coupled with Time-of-Flight Mass Spectrometry

**DOI:** 10.1155/2013/763213

**Published:** 2013-12-05

**Authors:** Jida Zhang, Hao Cai, Gang Cao, Xiao Liu, Chengping Wen, Yongsheng Fan

**Affiliations:** ^1^College of Basic Medical Science, Zhejiang Chinese Medical University, Hangzhou 310053, China; ^2^College of Pharmacy, Nanjing University of Chinese Medicine, Nanjing 210023, China; ^3^Research Center of TCM Processing Technology, Zhejiang Chinese Medical University, Hangzhou 310053, China

## Abstract

Ultraperformance liquid chromatography coupled with time-of-flight mass spectrometry (UPLC-QTOF/MS) based on a chemical profiling method was applied to rapidly evaluate the chemical consistency between sun-dried and sulfur-fumigated Radix Paeoniae Alba. By virtue of the high resolution, high speed of UPLC, and the accurate mass measurement of TOFMS coupled with reliable MarkerLynx software, five newly assigned monoterpene glycoside sulfonates were found and identified in sulfur-fumigated Radix Paeoniae Alba samples. This method could be applied for rapid quality evaluation of different kinds of sulfur-fumigated Radix Paeoniae Alba among commercial samples.

## 1. Introduction

Sulfur-fumigation processing is one of the traditional preservative approaches for most food, agricultural product, and herbal medicine. Moreover, some herbal farmers even sprinkle sulfur powder onto the herbs to infiltrate sulfur into the herbs. The sulfur-fumigation process uses sulfur dioxide, obtained from the heating of sulfur, for the fumigation of herbal medicines as well as processed drugs, for the purposes of torrefaction, sterilization, mildew proofing, insect prevention, and bleaching [1, 2]. However, the medicinal properties and chemical profiles are likely to be changed after the sulfur-fumigation process. Besides, residual sulfur dioxide in herbal medicine is harmful to human health. According to some reports, a sulfur dioxide concentration of greater than 0.05% in the medicine can cause an unpleasant taste sensation [3]. Long-term administration of such sulfur-fumigated medicine may cause heavy metal intoxication in the body. Furthermore, excess sulfur dioxide in herbal medicine may cause a sore throat and stomachache, and more seriously it can even lead to a toxic reaction in the liver and kidneys [4, 5].

Radix Paeoniae Alba, derived from the dried root of *Paeonia lactiflora* Pall., is one of the oldest and most frequently used herbal medicines [6]. Radix Paeoniae Alba has been applied clinically in traditional Chinese Medicine to calm liver wind, relieve pain, nourish blood, regulate menstrual functions, and suppress sweating [7, 8]. Furthermore, Radix Paeoniae Alba contains significant amounts of monoterpenoid glucosides, tannins, phenolic acids, triterpenes, saponins, and other substances that are considered to be the biologically active components critical in many TCM formulas [9, 10]. Traditionally, the Radix Paeoniae Alba was dried naturally under sun or in the shade, but in recent decades, this practice has been replaced by sulfur-fumigation, a faster and cheaper method. Accumulated studies showed that sulfur-fumigation can induce chemical transformation of paeoniflorin, the main bioactive component of Radix Paeoniae Alba, into its artifact paeoniflorin sulfonates and consequently alter the bioactivities and pharmacokinetics of Radix Paeoniae Alba [11]. Whether the constituents of sulfur-fumigated Radix Paeoniae Alba changed or not is a very important issue referring to both efficacy and safety when applied in clinic. Development of a rapid and specific approach to determine the potential chemical changes is the key to the quality evaluation of sulfur-fumigated Radix Paeoniae Alba.

Ultraperformance liquid chromatography (UPLC) coupled with time-of-flight mass spectrometry (QTOF/MS) is a powerful hyphenated technique and has been used as a major tool for the quality assurance of herbal medicine and its preparations [12, 13]. Compared to conventional liquid chromatography, UPLC using short columns packed with 1.7-1.8 *μ*m porous particles holds enhanced retention time reproducibility, high chromatographic resolution, improved sensitivity, and increased operation speed.

In the present study, UPLC-QTOF/MS coupled with a chemical profiling approach was developed to investigate the influence of sulfur-fumigation on the quality of Radix Paeoniae Alba. Under the chromatographic and MS conditions, the significantly changed components, in particular those newly generated components in sulfur-fumigated Radix Paeoniae Alba, were identified or tentatively assigned by comparing their mass spectra with the LC-MS/MS library and/or tentatively assigned by matching empirical molecular formula with that of published compounds and/or elucidating quasimolecular ions and fragment ions referring to the available literature information. This method could be applied for rapid quality evaluation of different kinds of sulfur-fumigated Radix Paeoniae Alba among commercial samples.

## 2. Experimental

### 2.1. Chemicals, Solvents, and Herbal Materials

The reference sun-dried Radix Paeoniae Alba samples were acquired from the suppliers of Bozhou (Anhui, China). The identities of the collected reference sun-dried Radix Paeoniae Alba samples were authenticated to be the dried root of *Paeonia lactiflora* Pall. using morphological and histological methods according to Chinese Pharmacopoeia (version 2010) by an expert in the field. HPLC-grade acetonitrile, was obtained from Merck (Darmstadt, Germany). Deionized water was purified using the Milli-Q system (Millipore, Bedford, MA, USA); formic acid was of HPLC grade and was obtained from Honeywell Company (Morristown, New Jersey, USA). All other chemicals were of analytical grade and commercially available.

### 2.2. Liquid Chromatography

UPLC was performed with a Waters ACQUITY UPLC system (Waters Corp., MA, USA), equipped with a binary solvent delivery system, autosampler, and a PDA detector. The column was a Waters ACQUITY BEH C_18_  (100 mm × 2.1 mm, 1.7 *μ*m). The mobile phase consisted of (A) 0.1% (v/v) aqueous formic acid and (B) acetonitrile. The UPLC elution condition was optimized as follows: 2–15% B (0–8 min), 15–30% B (8–11 min), 30–50% B (11–13 min), 50–2% B (13–13.05 min), and 2% B (13.05–15 min). The detection wavelength was set at 270 nm, and the flow rate was at 0.5 mL·min^−1^. The temperatures of column and autosampler were maintained at 35 and 10°C, respectively, and the injection volume of sample was 2.0 *μ*L.

### 2.3. Mass Spectrometry

Mass spectrometry was performed on a Waters Xevo QTOF/MS system (Waters Corp., MA, USA), equipped with an electrospray ionization (ESI) source. The nebulization gas was set at 650 L·h^−1^. At temperature of 350°C, the cone gas was set at 50 L·h^−1^, and the source temperature was set at 120°C. Detection was performed in negative ion modes in the *m*/*z* range of 100–1000 Da, with an acquisition time of 0.3 s in centroid mode. The ESI conditions were as follows: capillary voltage 2500 V, cone voltage 30 V, source temperature 120°C, desolvation temperature 400°C, cone gas flow 50 L*·*h^−1^, and desolvation gas flow 800 L*·*h^−1^.

### 2.4. Accurate Mass Measurement

All MS data were acquired using the LockSpray to ensure mass accuracy and reproducibility. Mass axis was calibrated during the experiment by continuous infusion of a solution of 2 g·mL^−1^ leucine enkephalin in acetonitrile/water (50 : 50). The data were collected using MassLynx v4.1 software and analyzed using MassFragment (Waters Corp., Milford, MA, USA).

### 2.5. Sample Preparation

#### 2.5.1. Sulfur-Fumigated Radix Paeoniae Alba Samples

The sulfur-fumigated samples were prepared from the reference sun-dried Radix Paeoniae Alba samples, following procedures similar to that employed by farmers and wholesalers: 150 g of the reference sun-dried Radix Paeoniae Alba sample was wetted with 15 mL of water and then put to stand for 1.5 h; 15 g of sulfur powder was heated until burning; the burning sulfur and the wetted reference sun-dried Radix Paeoniae Alba sample were carefully put into the lower and upper layers of a desiccator, respectively. The desiccator was then kept closed for 5 h. After fumigation, the sulfur-fumigated Radix Paeoniae Alba sample was dried in a ventilated drying oven at 40°C for 5 h.

#### 2.5.2. Radix Paeoniae Alba Sample Solutions

The powder of sun-dried or sulfur-fumigated Radix Paeoniae Alba sample was precisely weighed (0.5 g) and extracted with 5 mL of 50% methanol in an ultrasonic bath (power 200 W, frequency 40 kHz) for 25 min and cooled at room temperature; then 50% methanol was added to compensate for the lost weight. The sample solution was centrifuged for 10 min and the supernatant was further diluted to a proper concentration and filtered through a 0.45 *μ*m filter membrane before being injected into the UPLC system for analysis.

### 2.6. Multivariate Statistical Analysis

All data were processed using the MarkerLynx application manager for MassLynx 4.1 software (Waters Corp., Milford, USA). The unsupervised segregation was checked by principal component analysis (PCA) using Pareto-scaled data. The first objective in the data analysis process is to reduce the dimensionality of the complex data set to enable easy visualization of any component clustering of the different groups of samples. Thus, the loading plot gives an indication of the components that most strongly influence the patterns in the score plot. From the loading plot of orthogonal to partial least squares discriminant analysis (OPLS-DA), various components could be identified as being responsible for the differentiation between sun-dried and sulfur-fumigated samples and were therefore viewed as potential chemical transformations.

## 3. Results and Discussion

### 3.1. Chromatographic and MS Conditions Development

**Figure 1:**
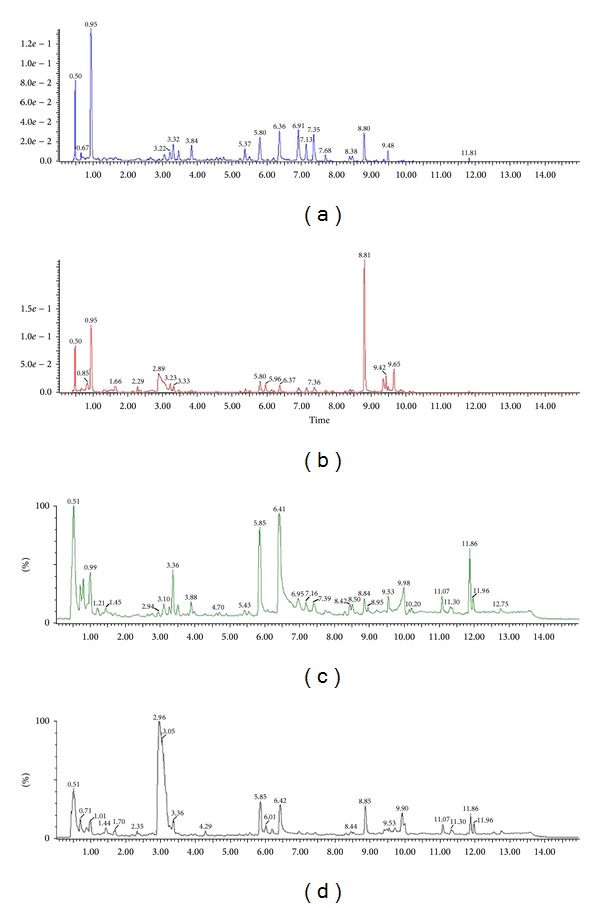
Representative chromatograms of sun-dried and sulfur-fumigated Radix Paeoniae Alba samples in negative ion mode. (a) UPLC chromatogram of sun-dried Radix Paeoniae Alba; (b) UPLC chromatogram of sulfur-fumigated Radix Paeoniae Alba; (c) total ion chromatogram of sun-dried Radix Paeoniae Alba; (d) total ion chromatogram of sulfur-fumigated Radix Paeoniae Alba.

In the present study, different kinds of UPLC columns and mobile phases were used to optimize the chromatographic and MS conditions. The BEH C_18_ column was chosen as stationary phase, which provided a relatively even distribution of the target analytes throughout the whole polarity range. This enabled us to substantially shorten the duration of chromatography to 15 min including equilibration. Different mobile phase compositions were tested: water-methanol, water-acetonitrile, 0.1% (v/v) aqueous formic acid–methanol, and 0.1% (v/v) aqueous formic acid–acetonitrile. As a result, the combination of 0.1% (v/v) aqueous formic acid–acetonitrile for the mobile phase gave the best separation. The gradient elution profile and MS conditions were optimized with respect to the separation of major peaks and the sensitivity of MS detector. Under the optimized chromatographic and MS conditions, the major components in sun-dried and sulfur-fumigated Radix Paeoniae Alba samples were well separated and detected within 15 min. The representative chromatograms monitored by UPLC-QTOF/MS are shown in Figure 1.

### 3.2. Multivariate Statistical Analysis and Chemical Consistency Evaluation

**Figure 2:**
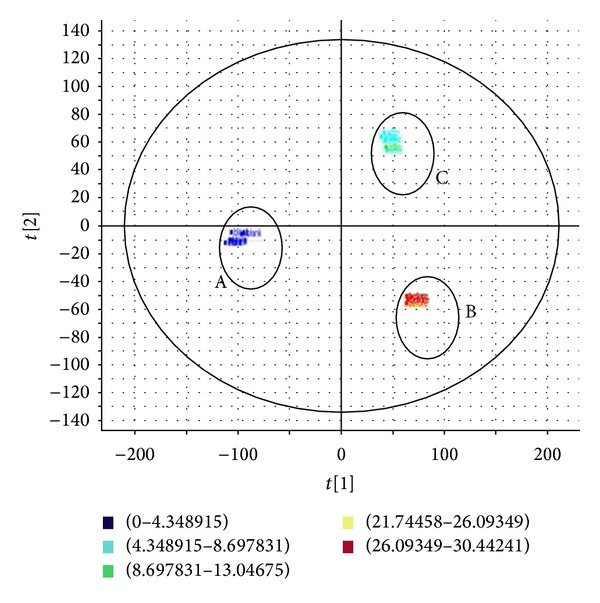
PCA score plot of sun-dried and sulfur-fumigated Radix Paeoniae Alba samples. A:  blank; B:  sun-dried Radix Paeoniae Alba samples; C:  sulfur-fumigated Radix Paeoniae Alba samples.

Unsupervised principal component analysis (PCA) and supervised orthogonal partial squared discriminant analysis (OPLS-DA) were performed to compare the difference between sun-dried and sulfur-fumigated Radix Paeoniae Alba samples. After Pareto scaling with mean centering, the data from both negative ion modes were displayed as score plot (Figure 2). The score plot showed that the determined samples clearly clustered into three groups, that is, the blank, the sun-dried, and the sulfur-fumigated Radix Paeoniae Alba, indicating that the sulfur-fumigation caused changes in the composition and/or content of components in Radix Paeoniae Alba.

**Figure 3:**
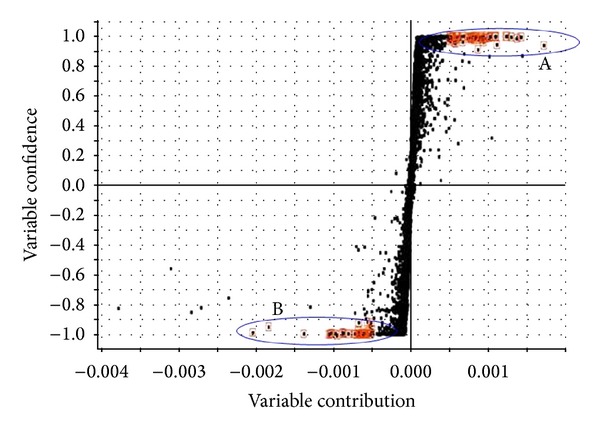
OPLS-DA/S-plot of sun-dried and sulfur-fumigated Radix Paeoniae Alba samples obtained using Pareto scaling with mean centering. A: sun-dried Radix Paeoniae Alba samples; B: sulfur-fumigated Radix Paeoniae Alba samples.

To find out potential chemical markers contributing to the significant difference between sun-dried and sulfur-fumigated Radix Paeoniae Alba samples, the extensive statistical analysis was performed to generate S-plot (Figure 3). In the S-plot, each point represents an ion *t*_*R*_-*m*/*z* pair; the *x*-axis represents variable contribution: when the distance of the ion *t*_*R*_-*m*/*z* pair points is farther from zero, the ion has more contribution to the difference between the two groups; the *y*-axis represents variable confidence: when the distance of the ion *t*_*R*_-*m*/*z* pair points is farther from zero, the ion has higher confidence level for the difference between two groups. Thus, the *t*_*R*_-*m*/*z* pair points at the two ends of “S” represent characteristic markers with the most confidence to each group. According to the S-plot, five ions at the bottom left corner of “S” were the ions contributing most to the difference between sun-dried and sulfur-fumigated Radix Paeoniae Alba. It was found that ions A (*t*_*R*_ 1.709 min, *m*/*z* 559.1106), B (*t*_*R*_ 2.338 min, *m*/*z* 589.1216), C (*t*_*R*_ 3.002 min, *m*/*z* 543.1139), D (*t*_*R*_ 6.016 min, *m*/*z* 695.1207), and E (*t*_*R*_ 9.863 min, *m*/*z* 647.1406) were detected in sulfur-fumigated Radix Paeoniae Alba but not found in the sun-dried sample, which suggested that during the storage and marketing process, in order to preserve its white appearance, Radix Paeoniae Alba was often nonofficially fumigated with toxic sulfur dioxide gas, which is generated by burning sulfur. This nonofficial postharvest handling method was recently revealed leading to the conversion of the main component paeoniflorin into its sulfonate derivative paeoniflorin sulfonate.

### 3.3. Identity Assignment and Confirmation of the Significantly Transformed Components

**Figure 4:**
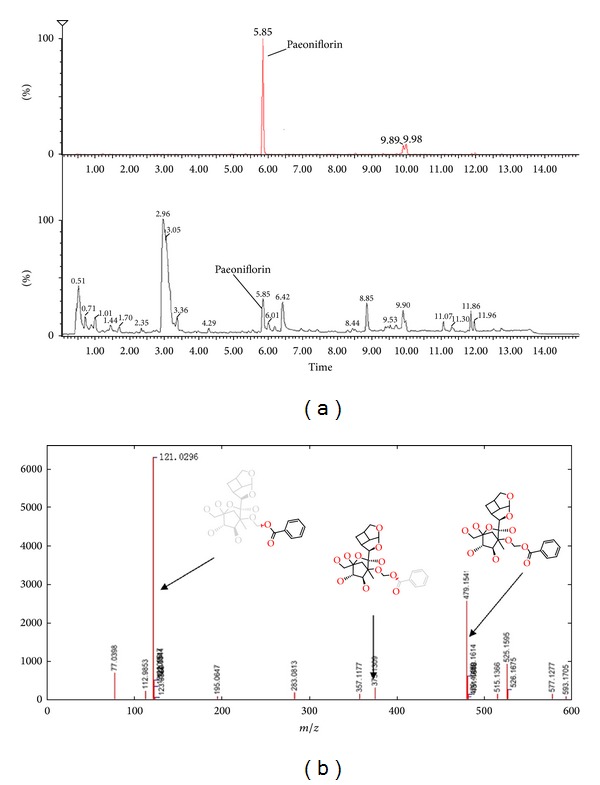
Chromatograms and fragmentations and mode assignments of paeoniflorin. (a) Radix Paeoniae Alba sample; (b) paeoniflorin assay standard.

**Figure 5:**
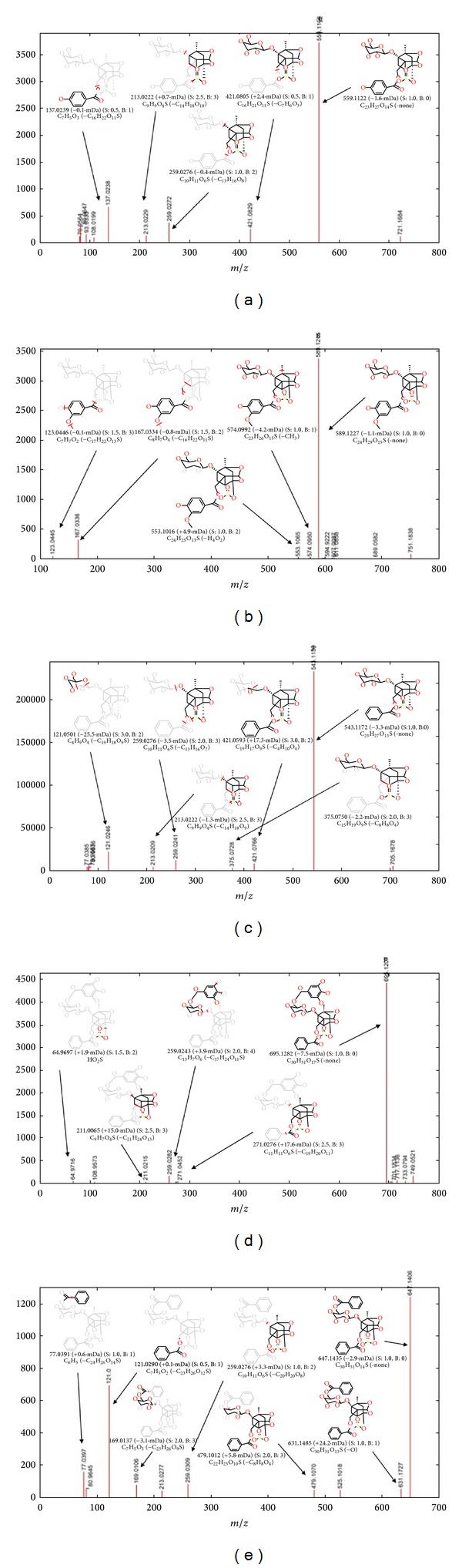
Fragmentations and mode assignments of five monoterpene glycoside sulfonate derivatives. (a) Oxypaeoniflorin sulfonate; (b) mudanpioside E sulfonate; (c) paeoniflorin sulfonate; (d) galloylpaeoniflorin sulfonate; (e) benzoylpaeoniflorin sulfonate.

In the present study, significant differences in their chemical profiles were found between sun-dried and sulfur-fumigated Radix Paeoniae Alba samples. Five monoterpene glycoside sulfonate derivatives were detected in sulfur-fumigated Radix Paeoniae Alba samples according to the present chromatographic and MS conditions. The details of the five identified components are summarized in Table 1. Five monoterpene glycoside sulfonate derivatives were newly detected and identified along with oxypaeoniflorin sulfonate, paeoniflorin sulfonate, mudanpioside E sulfonate, benzoylpaeoniflorin sulfonate, and galloylpaeoniflorin sulfonate by MassFragment system and MarkerLynx system. MarkerLynx is a peak detection algorithm, where each mass number is analyzed separately in search of peaks. The software MarkerLynx has been used to process the complex data quickly and reliably. In this paper, we firstly applied the MarkerLynx to the analysis of the structure of paeoniflorin in Radix Paeoniae Alba samples. This software is a repeatable and reliable analytical method when we should compare the MS data generated by using paeoniflorin assay standard. The chromatograms and fragmentations and mode assignments of paeoniflorin assay standard are shown in Figure 4, and the fragmentations and mode assignments of those five monoterpene glycoside sulfonate derivatives are shown in Figure 5. As sulfur-fumigation can cause chemical transformation of Radix Paeoniae Alba, the bioactivities and toxicities of sulfur-fumigated Radix Paeoniae Alba and five monoterpene glycoside sulfonate derivatives need further investigation.

## 4. Conclusion

In this paper, chemical consistency between sun-dried and sulfur-fumigated Radix Paeoniae Alba samples was rapidly evaluated by UPLC-QTOF/MS based on chemical profiling approach to guarantee clinical safety. This method was also developed to reveal chemical transformation of main compounds in Radix Paeoniae Alba during sulfur-fumigation process. The results showed that there were obvious differences in chemical components between sun-dried and sulfur-fumigated Radix Paeoniae Alba samples following the same dosage ratio. The established method should be useful for assessing the quality of herbal medicines.

## Figures and Tables

**Table 1:** Details of the five identified monoterpene glycoside sulfonate derivatives.

No.	*t*_*R*_ (min)	[M-H]^−^/(*m/z*)	Molecular formula	Assigned identity	Source sample
1	1.709	559.1106	C_23_H_28_O_14_S	Oxypaeoniflorin sulfonate	Sulfur-fumigated sample
2	2.338	589.1216	C_24_H_30_O_15_S	Mudanpioside E sulfonate	Sulfur-fumigated sample
3	3.002	543.1139	C_23_H_28_O_13_S	Paeoniflorin sulfonate	Sulfur-fumigated sample
4	6.016	695.1207	C_30_H_32_O_17_S	Galloylpaeoniflorin sulfonate	Sulfur-fumigated sample
5	9.863	647.1406	C_30_H_32_O_14_S	Benzoylpaeoniflorin sulfonate	Sulfur-fumigated sample
